# Virtual wards for people with frailty: what works, for whom, how and why—a rapid realist review

**DOI:** 10.1093/ageing/afae039

**Published:** 2024-03-14

**Authors:** Maggie Westby, Sharea Ijaz, Jelena Savović, Hugh McLeod, Sarah Dawson, Tomas Welsh, Hein Le Roux, Nicola Walsh, Natasha Bradley

**Affiliations:** The National Institute for Health and Care Research, Applied Research Collaboration West (NIHR ARC West), University Hospitals Bristol NHS Foundation Trust, Bristol BS1 2NT, UK; Bristol Medical School, University of Bristol, Bristol BS8 2PS, UK; The National Institute for Health and Care Research, Applied Research Collaboration West (NIHR ARC West), University Hospitals Bristol NHS Foundation Trust, Bristol BS1 2NT, UK; Bristol Medical School, University of Bristol, Bristol BS8 2PS, UK; The National Institute for Health and Care Research, Applied Research Collaboration West (NIHR ARC West), University Hospitals Bristol NHS Foundation Trust, Bristol BS1 2NT, UK; Bristol Medical School, University of Bristol, Bristol BS8 2PS, UK; The National Institute for Health and Care Research, Applied Research Collaboration West (NIHR ARC West), University Hospitals Bristol NHS Foundation Trust, Bristol BS1 2NT, UK; Bristol Medical School, University of Bristol, Bristol BS8 2PS, UK; The National Institute for Health and Care Research, Applied Research Collaboration West (NIHR ARC West), University Hospitals Bristol NHS Foundation Trust, Bristol BS1 2NT, UK; Bristol Medical School, University of Bristol, Bristol BS8 2PS, UK; Bristol Medical School, University of Bristol, Bristol BS8 2PS, UK; RICE – The Research Institute for the Care of Older People, Bath, UK; Royal United Hospitals Bath NHS Foundation Trust, Bath, UK; Churchdown Surgery, Parton Rd, Churchdown, Gloucester GL3 2JH, UK; NHS England and NHS Improvement South West, Somerset, UK; One Gloucestershire Integrated Care System Quality Improvement, Gloucester, UK; The National Institute for Health and Care Research, Applied Research Collaboration West (NIHR ARC West), University Hospitals Bristol NHS Foundation Trust, Bristol BS1 2NT, UK; Centre for Health & Clinical Research, University of the West of England, Bristol BS16 1DD, UK; School of Nursing and Midwifery, Queens University Belfast, Belfast BT7 1NN, UK

**Keywords:** virtual ward, frailty, multidisciplinary, proactive care, acute care, qualitative research, older people

## Abstract

**Background:**

Virtual wards (VWs) deliver multidisciplinary care at home to people with frailty who are at high risk of a crisis or in crisis, aiming to mitigate the risk of acute hospital admission. Different VW models exist, and evidence of effectiveness is inconsistent.

**Aim:**

We conducted a rapid realist review to identify different VW models and to develop explanations for how and why VWs could deliver effective frailty management.

**Methods:**

We searched published and grey literature to identify evidence on multidisciplinary VWs. Information on how and why VWs might ‘work’ was extracted and synthesised into context-mechanism-outcome configurations with input from clinicians and patient/public contributors.

**Results:**

We included 17 peer-reviewed and 11 grey literature documents. VWs could be short-term and acute (1–21 days), or longer-term and preventative (typically 3–7 months). Effective VW operation requires common standards agreements, information sharing processes, an appropriate multidisciplinary team that plans patient care remotely, and good co-ordination. VWs may enable delivery of frailty interventions through appropriate selection of patients, comprehensive assessment including medication review, integrated case management and proactive care. Important components for patients and caregivers are good communication with the VW, their experience of care at home, and feeling involved, safe and empowered to manage their condition.

**Conclusions:**

Insights gained from this review could inform implementation or evaluation of VWs for frailty. A combination of acute and longer-term VWs may be needed within a whole system approach. Proactive care is recommended to avoid frailty-related crises.

## Key Points

- This rapid realist review covers how, why and in which contexts virtual wards may be effective, and informs service planning.
- Frailty virtual wards may provide short-term acute care for frailty crisis or longer-term proactive care to prevent a crisis.
- Evidence-based theories show how virtual ward components combine to deliver frailty care and empower patients and caregivers.
- A whole-system approach is key to good virtual ward frailty management, involving continuity of care (referral and discharge).
- Sustainability of virtual wards requires a focus on proactive care to prevent frailty crises and reduce hospital admission.

## Introduction

Frailty is a dynamic and multidimensional syndrome associated with age-related decline in multiple physiological systems [1–3]. People with frailty are vulnerable to unpredictable deteriorations in health, and minor stressor events can lead to medical crises, from which the person does not fully recover [1]. Medical crises in people with frailty are associated with poorer outcomes [4], and can lead to increased care dependency and acute hospital admission [1, 5, 6].

The UK has an ageing population with an increasing prevalence of frailty [1, 7, 8], and the need for innovation in frailty management is recognised [5, 9]. People with frailty form a diverse group, requiring different levels of support from health and social care. Their dependence on others for activities of daily living may put them at a risk of hospital admission and delayed discharge because of a lack of community services [10].

Delivering services to support people living with frailty requires a multidisciplinary team (MDT) that can provide an iterative, tailored, whole-person approach to diagnosis, assessment and treatment, aiming to promote function and independence, intervene with crises, and prevent exacerbations [1, 5, 11–13]. Virtual ward (VW) models combine components of care under a common scheme, delivering multidisciplinary care to patients in their own homes, aiming to mitigate their risk of unplanned hospitalisation.

VWs for people living with frailty or long-term conditions were first introduced in the 2000s in the UK [14–17]. Further development of frailty VWs was influenced by seminal work from Ireland by Lewis et al. [16] Building on this earlier work and experience of COVID-19 VWs [18], NHS England (NHSE) issued guidance on short-stay (a few days) VWs for patients with ‘acute exacerbations of conditions related to frailty’, with planned roll-out [19]. The term, ‘virtual ward’ is, however, used to cover a variety of models.

Evidence of VW effectiveness is limited. Five studies [15, 20–23] and one systematic review [24] that compare VWs with usual care (one UK-based [15]) report inconsistent findings. Suggested explanations for poor effectiveness include failure in MDT functioning [15], indicating there may be crucial mechanisms by which VWs ‘work’ to improve patient outcomes.

### Rationale

Multidisciplinary VWs are being introduced, driven by the limited availability of acute hospital beds, and the desire to treat people in their own homes, but initiatives are complex and diverse. As part of a larger programme investigating combined interventions for people with frailty in the UK, our research questions sought, first, to determine what multidisciplinary VW models were in operation in the UK; and second, to understand how and why they may work (or not) within their specific contexts (rather than whether they work), focusing particularly on frailty VWs.

Rapid realist reviews are a suitable framework for answering such (how and why) questions because they incorporate a range of evidence and account for contextual variation within and across VW models. Rapid realist reviews allow investigation of a defined topic area to inform policy by identifying key components of services that should be customised to achieve effectiveness [25].

Our intention to focus on UK evidence was to reduce the large variations potentially introduced by different health and social care systems worldwide.

This rapid realist review aimed to synthesise relevant evidence, producing initial programme theories explaining ‘what works, for whom, and in what circumstances?’ that can be tested empirically in future work [26].

## Methods

Preliminary scoping of the literature informed development of the review protocol, including our definition of VWs. We conducted two rounds of literature searching and three rounds of realist synthesis. Stakeholders were engaged in both. For further details, see below and Appendix I [25, 27].

### Inclusion criteria

We used a broad definition of (multidisciplinary) VWs, limited to three essential components (Box 1; Appendix II) [17, 24, 28]:

- Care is provided to the patient in their own home in the community.
- A multidisciplinary team makes decisions/plans care remotely from the patient.
- The MDT provides oversight of patient care.

‘Virtual’ refers to the way MDTs plan each patient’s care, remote from the patient—as opposed to remote patient monitoring [28, 29] (Appendix II). We placed no restrictions on the timelines and aims of the VWs, because we intended to identify different models.

Other inclusion criteria were: (i) people with frailty or multi-morbidities; (ii) set in the UK; and (iii) relevance, i.e. whether data can contribute to theory development and refinement (rapid realist review).

We accepted authors’ definitions of frailty, and included populations with multimorbidities where frailty was not reported. Frailty and multimorbidity, although different clinical conditions, are interrelated and both are predictors and outcomes of each other [3] (Box 1).

**BOX 1:** Definitions

**CMO:** 

- **Context:** backdrop of the intervention and variations of this across sites, which existed before the VW implementation and are outside of the mandate of service redesign (e.g. policy, staff skills, IT systems).
- **Mechanism:** reasoning of stakeholders in response to resources offered by the intervention (e.g. trust and motivation to act).
- **Outcome:** includes intended and unintended outcomes of interest, such as: hospital admissions, safety, clinical outcomes, resource use, patient and caregiver satisfaction, etc.
- **CMO Configurations (CMOCs):** propositions explaining how the interaction between contexts and mechanisms can lead to outcomes of the intervention (i.e. VWs for frailty).

**Virtual ward:** cares for patients in their own homes (in the community) and there is an MDT that makes decisions/plans patient care remotely (virtually) and the MDT provides oversight and integration of patient care:

- The virtual part of a VW is the way multi-disciplinary teams of health and care professionals plan each patient’s care, using digital technology to help them meet.

**Multidisciplinary team (MDT):** people with frailty—a multidimensional condition—require care tailored to their needs from a multidisciplinary, integrated health and social care team. This MDT may include primary care, community care and secondary care professionals, alongside social workers, pharmacists, physiotherapists, mental health professionals, voluntary sector staff, etc.

**Frailty:** a state of increased vulnerability to unpredictable deterioration in health, associated with an age-related decline in multiple physiological systems, which puts the person at high risk of frailty crises. In people with severe/moderate frailty, crises may be triggered even by minor events, leading to subsequent adverse outcomes and acute hospital admission:

- Frailty crises include severe falls, delirium, and sudden immobility.
- Triggers can be something small, such as a minor infection or injury, constipation, new medication, a visit to A&E (or can be bigger events/acute illnesses)

**‘Acutely unwell’:** person with frailty who is either at high risk of a frailty crisis (and requiring preventative treatment) or already in-crisis (and requiring reactive treatment initially).

For theory building, any type of evidence and research design was eligible, from peer-reviewed papers to grey literature, including service case reports, videos and blogs.

We intended to restrict the review searches to the UK. However, alongside limited UK evidence, the first main search identified the papers of Lewis et al., reporting work from Ireland that operated within similar population demographics, with some similarities in healthcare provision (a public-private system), and we deemed it relevant to include this work because it had been influential for frailty VWs in the UK. Four further Irish documents did not meet the inclusion criteria.

We excluded VWs in care homes, children, people with COVID-19 or a single condition (e.g. cystic fibrosis).

### Searching and selection of documents

Literature searching was done iteratively within two rounds (see Appendix III for the full search strategy and Figure 2): for the rapid realist review, the first main search (to 8 November 2021) was to find relevant ‘core’ documents from which we extracted if-then-because statements in an iterative way. Then after synthesising the data, we conducted an updated and revised search (to 27 June 2022) to find further evidence and to address evidence gaps identified by stakeholders, thereby refining and expanding on our original programme theories.

During initial literature scoping for protocol development, we noted debate around VWs being a distinct model of care compared with hospital-at-home. Following advice from topic experts, we initially agreed the ‘hospital-at-home’ in scoping documents did not fit our VW definitions, and therefore excluded studies reporting hospital-at-home. However, during engagement meetings, stakeholders shared documents not found by either scoping or first round searches, which they considered pertinent. This included a hospital-at-home model that met our frailty VW definition [30]. We then broadened the search terms to include hospital-at-home variations and removed the exclusion criterion. Hospital-at-home models were eligible if they met our definition of VWs (Appendix II).

In both rounds, we searched Ovid multi-file databases (MEDLINE, Embase, PsycINFO), using terms relating to multidisciplinary teams, remote/virtual care, frailty/multimorbidities/older people and the UK. In the second search, we added the extra terms, examined reference lists of systematic and other realist reviews, searched for grey literature and conducted a forward citation search using included documents.

Title and abstracts, and full texts were screened in duplicate for the first search, with discrepancies resolved by a third reviewer, identifying relevant and information-rich ‘core’ documents. Following the second search, results were single screened in reverse chronological order, back to 2018 and full papers assessed in duplicate.

### Data extraction and synthesis

Eight ‘core’ documents from the first search were reviewed by three authors, who extracted data as ‘if-then-because’ statements that captured relevant causal insights; we grouped these statements thematically into 21 topic areas. These topics informed the first stakeholder meetings.

Next, we developed ‘context-mechanism-outcome configurations’ (CMOCs) (Box 1). Synthesis was iterative: preliminary CMOCs were articulated, using the extracted data and stakeholder engagement, and then elaborated and refined from further included documents. This process (Appendix IV) resulted in 12 CMOCs (Appendix V), which we summarise below, alongside implications for practice.

We assessed the trustworthiness, plausibility and coherence of the data and checked our findings with stakeholders. We did not formally appraise the evidence quality using checklists or assess confidence in the evidence because these tools could not capture the different ways that documents contribute to a programme theory, and we were generating theories, rather than testing them [31].

### Stakeholder engagement

Stakeholders were recruited through our organisation’s patient and public involvement (PPI) programme. They included one GP with frailty expertise and COVID VW experience, three carers of people with frailty, three patients (one with frailty, and one with COVID VW experience), a general practice administrator with VW experience, and two geriatricians known to the team, who had frailty VW experience.

**Figure 1:**
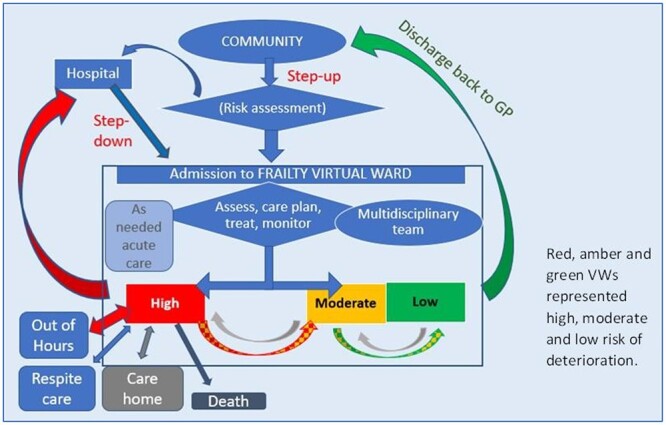
Patient pathway within a VW model.

We engaged with stakeholders at two timepoints. First, we developed and presented the patient pathway within a VW (Figure 1) to one clinician and two PPI contributors, requesting feedback, and facilitated discussion on the topic areas of the if-then-because statements. This generated further statements, based on stakeholder experience, derived from the meeting transcripts.

Meanwhile, building on VWs for COVID-19 [18], NHSE had issued guidance on short-stay (a few days) VWs for patients with ‘acute exacerbations of conditions related to frailty,’ with planned roll-out of these [19]. Therefore, in the second round of stakeholder engagement, we presented draft CMOCs and made comparisons with NHSE guidance.

The second round involved three clinicians and five PPI contributors. Based on their feedback and the NHSE guidance, we broadened the review to acute, hospital-at-home models, provided they met our VW inclusion criteria.

## Results

We describe document characteristics, the different VW models, and summarise 12 CMOCs under three main themes. Full details of the CMOCs are in Appendix V.

### Document characteristics

**Figure 2:**
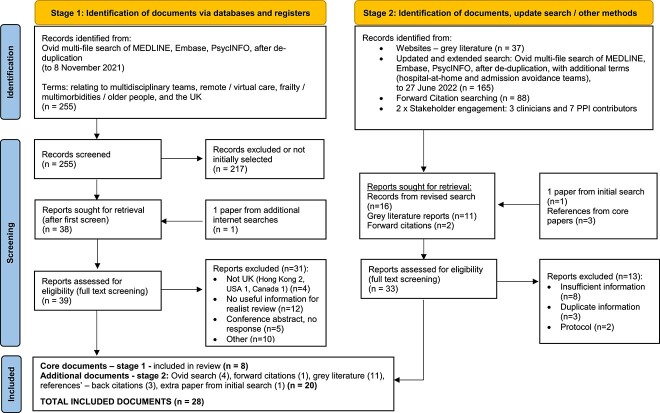
Flow diagram.

The search process is depicted in Figure 2. Details of included documents are in Appendix VI. We included eight core documents from the first search [16, 17, 32–37] and 20 documents from stage 2 (nine peer-reviewed [15, 30, 38–44] and 11 grey literature [6, 28, 29, 45–52]). Ten documents report on four studies [15–17, 29, 30, 36–38, 44, 49].

Thirteen documents specifically included people with frailty; [6, 16, 28–30, 36, 37, 43–45, 49, 50, 52] four described patients as ‘frail’ or measured frailty; [35, 41, 42, 46] eight included people with at least one chronic condition with high risk prediction scores for hospitalisation; [15, 17, 32, 33, 38, 39, 47, 51] two had implied chronic conditions and complex needs; [34, 48]; one listed urgent care needs [40]. Most studies were conducted over 5 years ago, and four were in 2020–2022 [28, 35, 45, 49].

### VW models

VWs provide care at home for people with frailty or chronic conditions at high risk of hospital admission. We distinguished two main VW models: model 1—longer-term (more than 3 weeks, typically 3–7 months) with mainly proactive care for people at high risk of a crisis, and model 2—short-term VWs (1–21 days) with mainly reactive care for those already in-crisis (Table 1, Box 1).

**Table 1:** VW models

	Model 1 (longer-term VWs)		Model 2 (short-term VWs)
	Model 1a (long-term conditions and predicted high risk of hospital admission)	Model 1b (frailty and at high risk of crisis)	Model 2 (frailty and already in crisis)
**Intention**	Reduce acute hospital admissions for people with chronic conditions at high risk of admission, living at home	Optimise medical, functional, psychological support to reduce risk of crisis/deterioration, and reduce acute hospital admission	Acute hospital level care at home, reduce hospital admission and expediate hospital discharge
**Duration**	3–7 months average	90 days average	1–21 days
**Population**	Not specifically people with frailty; patients tend to be older with multiple chronic diseases (many of whom would also have frailty), identified to be at high risk of hospital admission	People with moderate/severe frailty (frailty tools or with impaired function/loss of autonomy in activities of daily living), identified as at high risk of a crisis or frailty deterioration	People with frailty who are in-crisis, who have been in-crisis or are near to being in-crisis
**Patient selection method**	Using risk prediction tools for hospital admission. Usually referral from the community	Using frailty severity tools, risk of hospital admission score and clinical judgement. Mainly community referral, also post-hospital discharge	Using frailty severity tools and clinical judgement. Either referral from the community or post-hospital discharge
**Treatment offered**	Proactive (for long-term chronic conditions). May be separated by the risk level into different VWs (e.g. red, amber, green VWs)	CGA: reactive as needed, but mainly proactive to stabilise, and reduce the risk of medical crises in people with frailty. May be separated by risk into different VWs	CGA: reactive for those in/near crisis, then may start proactive care and monitoring if time before discharge to GP. Continuation of proactive care with GP after discharge from VW. Not usually separated by severity
**Alternative care (if no VW)**	Usual care in the community (patients at risk of hospital admission)	Usual care in the community (patients at risk of hospital admission)	Acute inpatient care
**Discharge criteria and timing**	Discharge criteria (e.g. may be based on clinical decision or reduction in risk score). Goals in the treatment plan have been met, or the VW no longer adds value to the patient’s situation, or the patient no longer wants to participate.	Discharge criteria based on frailty stability (e.g. defined in terms of eating and drinking, cognitive status, activities of daily living, emotional/psychological state, no events for 30 days). Discharge by MDT once considered stable with no events for 4–6 weeks	Timing and criteria of discharge unclear. Appears to be when acute events have been resolved. Likely to require GP to continue/start CGA after discharge

Fifteen documents describe longer-term VWs [15–17, 28, 32–34, 36–39, 41, 43, 50, 51] ten short-term VWs, [29, 30, 35, 40, 42, 44, 45, 47, 49, 52], two both models [6, 46] and one was unclear [48].

Originally, longer-term VWs (e.g. [17], model 1a) were intended to reduce acute hospital admissions by proactively treating older people with chronic conditions at a high risk of admission. Patient selection was usually based on risk prediction modelling, aligning with UK policies for caring for vulnerable people [53].

Subsequent VWs focussed on frailty (e.g. [6, 16, 28, 50]; model 1b) reflecting 2016–17 changes in UK policy and emphasis [2, 54]. VWs aimed to assess and stabilise people living with frailty at high risk of a crisis, exemplified by Irish VWs [16], which influenced development in the UK, including NHSE guidance [19]. Reactive care was first offered to alleviate any acute exacerbations, before focusing on proactive care to reduce risk of future crises, for example, based on the Comprehensive Geriatric Assessment (CGA) [55].

Longer-term VWs are alternatives to usual care in the community for people at high risk of exacerbations. In some VWs, a traffic light system (red/amber/green) is used to prioritise assessment, monitoring, and intervention according to risk of deterioration [16, 17].

Short-term VWs admit people with frailty already in-crisis or very near to a crisis (e.g. [30], model 2), offering, principally, acute reactive care. Proactive care may be started in the VW if there is time, alongside planning for continuity after discharge to primary care. Short-term VWs are alternatives to inpatient hospital treatment. NHSE guidance on frailty VWs is based on this model [19].

Both models admit patients who are frail and ‘acutely unwell’ (Box 1), and aim to reduce system burden and improve frailty care. One is better suited for preventing crises, the other for in-crisis management. Both models aim to prevent hospital admission, either by preventing the crisis from occurring or by treating the crisis in the community instead of hospital admission.

Boundaries between models were sometimes blurred, for example, in one longer-term VW, patients with frailty who became ‘unstable’ were admitted to hospital; [36] one short-term VW additionally aimed to be proactive; [49] and some VWs were difficult to classify (e.g. [34, 39, 42, 43, 47, 48]). Two studies described both a short-term reactive ward and a longer-term proactive ward, potentially working in tandem [6, 46](South Sefton) (Appendix VI).

### CMOC Theme 1: VW building blocks (Table 2)

CMOCs in this section describe underlying structures essential for VW operation. They include common standards agreements, information sharing processes, MDT composition and co-ordination, and MDT meetings (or ‘Virtual Ward Rounds’). These CMOCs are not limited to a particular model of VW, and may also apply to non-frailty multidisciplinary VWs.

**Table 2:** Summary of CMOCs for Theme 1: VW building blocks

CMOC and sources	CMOC descriptions for Theme 1
**CMOC1: common standards agreements** Sources: 11 documents [6, 15, 17, 32, 35, 39, 41–44, 46], three clinicians (3/11 short-term, 4/11 non-frailty, 1/11 Ireland)	Common standards agreements are developed amongst different providers and specialities to meet their legal and regulatory requirements [Context]. Common standards agreements cover topics such as patient eligibility, assessment procedures, care documentation, data protection, safeguarding and discharge. Agreements suit the working practices and cultures of the different teams. There is transparency about the purpose and processes of the VW, offering clarity on role expectations, encouraging confidence that the VW will function and not put patients at risk [Mechanism]. Operational agreements formalise collaboration, underscore communication and teamwork between professionals, and facilitate effective decision-making and case management, leading to improved efficiency [Outcome].
**CMOC2: information sharing processes** Sources: 9 documents [15, 17, 32–34, 39, 43, 44, 47], one clinician, one PPI contributor (2/9 short-term, 7/9 non-frailty, 0/9 Ireland)	Progress towards IT integration and trust amongst providers allows information sharing and ‘real-time’ data management processes to be established amongst different organisations, both within and external to the VW [Context]. Professionals gain confidence from an accurate ‘whole system’ view of the patient record, when needed. Patient and caregivers appreciate not repeating themselves; and may feel reassured that their clinical team is well informed [Mechanism]. Decisions are better informed and timelier, and patient management is improved because care processes and access to interventions are streamlined [Outcome].
**CMOC3: MDT composition and coordination** Sources: 18 documents [6, 15–17, 28, 29, 32, 33, 35, 38, 41–45, 47, 50, 51], one clinician (6/18 short-term, 7/18 non-frailty, 2/18 Ireland)	Frailty management expertise is disparate across multiple teams, including primary care, community care, and speciality frailty clinicians. Key recognisable professionals can ‘champion’ working together in the VW [Context]. Professionals trust the model can provide safe and personalised care for patients at home. Effective VW co-ordinators facilitate teamwork, organise task sharing and liaise with both patient/caregivers and external organisations. All parties feel secure in the model. Professionals are willing and able to participate, taking a shared approach to tasks and problem-solving [Mechanism]. Patient management benefits from expertise and skills from different specialities and organisations. Team composition and coordination improves patient access to a range of interventions. Unnecessary or duplicated effort is reduced [Outcome].
**CMOC4: MDT meetings** Sources: 13 documents [6, 15–17, 32, 33, 38, 41–45, 51], one clinician (3/13 short-term, 6/13 non-frailty, 1/13 Ireland)	The aims and implementation of the VW model motivates different teams and disciplines to work together. Regular, well-attended meetings facilitate MDT function, provided the professionals involved have sufficient capacity in their workload [Context]. The MDT meets to discuss patients and plan their care, usually via technology, sometimes in-person. Meetings are forums for communication and link specialist clinicians and the care teams providing hands-on care. VW professionals perceive meetings are worthwhile and participate in collaborative problem-solving [Mechanism]. Collaboration improves holistic patient management, enhancing the effectiveness and efficiency of decision-making across different disciplines and providers. Supportive communication and task-sharing provides learning and upskilling opportunities for staff [Outcome].

#### Implications for VW operation

Sufficient motivation and co-operation amongst the teams involved are needed to develop and successfully introduce common standards agreements [15, 16, 32, 35, 42, 46]. These may need review and revision as the VW becomes more established [6, 43] [Clinician].

Similarly, introducing effective IT integration requires perseverance and collaboration amongst organisations [15, 32–34, 43, 44, 47]. Ineffective information sharing can mean duplication of effort and ‘silo working’ [17, 33]—frustrating for both staff and patients—and may impede the timeliness and/or appropriateness of decision-making [33, 34, 39, 43].

Team composition varies according to the aims of the particular VW model and local patient need. It may include geriatricians, physiotherapists, pharmacists, social workers, mental health professionals, voluntary sector, community organisations and other clinical specialities (e.g. cardiology) [6, 16, 17, 28, 29, 32, 35, 41, 44]. The MDT usually meets remote from the patient, with decisions enacted by community teams [6, 16, 43]. The role of the VW co-ordinator is pivotal [16, 28, 38, 47, 50, 51]. Successful communication and care documentation are essential [6, 17, 32, 42]. Ideally, all professionals feel confident they will have accurate information when they need it, facilitating prompt, well-informed decisions on patient management [16, 29].

The VW facilitates shared learning across traditional role boundaries, enhancing collective capacity for patient care [35, 43, 45, 51]. Co-location of VW team members could increase their connectedness and joint working [6, 41]. However, poor understanding of VW aims could lead to role protectionism that undermines MDT functioning [33].

Effective MDT meetings are crucial for the VW to function as a forum to integrate and prioritise patient care [6, 16, 17, 43, 45, 51]. Online meetings can facilitate attendance and save time, but professionals involved must have time and capacity to attend [Clinician]. Disparity in attendance could delay decision-making and demotivate attendees [17, 32, 38].

### CMOC Theme 2: VW delivering the frailty patient pathway (Table 3)

CMOCs in this section concern how the VW can optimally deliver care for people with frailty who are acutely unwell. CMOCs could be directly (*) or indirectly (#) applicable to multidisciplinary non-frailty VWs. CMOCs comprise patient selection*, comprehensive assessment and evaluation^#^, medication management*, intensive case management* and proactive care^#^.

**Table 3:** Summary of CMOCs for Theme 2: VW delivering the frailty pathway

CMOC and sources	CMOC descriptions for Theme 2
CMOC5: patient selection* Sources: 13 documents [6, 15–17, 28–30, 32, 33, 39, 47, 50, 51], two clinicians (3/13 short-term, 7/13 non-frailty, 1/13 Ireland)	Against a backdrop of scarce resources, VWs select and prioritise appropriate patients. Under the GP contract, GPs use frailty risk tools to identify people with frailty in the community, who are either in-crisis or nearing a ‘tipping point’ into crisis [Context]. Selection, informed by clinician judgement and frailty risk tools, or hospital risk prediction tools, prioritises patients to the VW, giving a coherent rationale for selection. Professionals perceive they can make a difference by working together to safely keep patients at home [Mechanism]. Selected patients receive timely and targeted management that could stabilise their condition or prevent a crisis, lowering the risk of unplanned hospitalisation and reducing length of stay if admitted [Outcome].
**CMOC6: comprehensive assessment and evaluation** ^**#**^ Sources: 7 documents [16, 17, 28, 29, 33, 44, 47] (3/7 short-term, 3/7 non-frailty, 1/7 Ireland)	Multidimensional needs of frailty require a holistic approach, such as the CGA [55]. MDT composition and functioning facilitate access to the interventions, specialists, and services required to address multidimensional frailty needs [Context]. Patients receive a holistic ‘assessment', usually face-to-face with the VW co-ordinator, using appropriate screening tools and goal setting. The VW co-ordinator and MDT prepare and enact a personalised management plan. The MDT feels confident in the information [Mechanism]. Patient’s needs are identified, appropriate interventions mobilised, and there is timely access to specialists and services. Reduced duplication of effort versus ‘siloed’ management may expedite access to interventions and could improve overall efficiency [Outcome].
CMOC7: medication management* Sources: 6 documents [15, 16, 34, 35, 44, 47], one clinician, one PPI contributor (3/6 short-term, 3/6 non-frailty, 2/6 Ireland)	Polypharmacy is common in people with frailty, and specialist medication management may be required [Context]. The VW enables a personalised medication review at home, allowing accurate reconciliation of prescribed and non-prescribed medications, and provides opportunity and extra time to discuss management, sensory impairment or side effects, explain any changes and respond to concerns. Patient and caregivers’ understanding of their treatment is improved [Mechanism]. Unnecessary polypharmacy is identified and resolved safely, potentially improving treatment adherence, reducing the risk of adverse events and treatment burden [Outcome].
CMOC8: intensive case management* Sources: 8 documents [6, 15–17, 32, 33, 44, 51], one clinician, two PPI contributors (1/8 short-term, 5/8 non-frailty, 1/8 Ireland)	People with frailty have complex health and social care needs. The VW brings together an effective ‘team of teams’ to deliver multidisciplinary care at home. Initially, interventions may be acute reactive treatment [Context]. In-person visits and monitoring provide accurate and timely information, and progress is reviewed regularly during MDT meetings, with a frequency determined by risk (e.g. using red/amber/green ratings) [15–17, 50]. The VW is well informed and responsive to patient needs. Patients/caregivers feel visible to the healthcare system in a way that feels safely supported [Mechanism]. The VW responds rapidly to changing patient needs and timely intervention is enacted. Through monitoring and review the VW determines when a patient is stable and ready for discharge to their GP [15, 16, 32, 33] [Outcome].
**CMOC9: proactive care** ^#^ Sources: 14 documents [6, 15–17, 28, 32, 33, 36, 39, 41, 44, 46, 47, 49], one clinician, one PPI contributor (3/14 short-term, 5/14 non-frailty, 2/14 Ireland)	Frailty is characterised by fluctuations in health, which can lead to frailty crises [Context]. Patients and caregivers receive proactive care to prevent a medical crisis (e.g. support for hydration, nutrition, and personal care; self-management strategies; advanced care planning; mental health; falls prevention; occupational health; physiotherapy and social support. VW professionals feel impactful in addressing potential issues and preventing future crises. Patients and caregivers feel supported and more confident in managing at home. Patients can be empowered to be active in their own care [17, 33, 46] [Mechanism]. Proactive care aims to stabilise a person living with frailty and supports patients/caregivers in living with frailty longer term. Preventing future deterioration and crises helps avoid acute hospital admission, and potentially improves quality of life and patient safety [Outcome].

These CMOCs are mainly informed by evidence related to 16 longer-term VWs, especially for patient selection, intensive case management and proactive care. Short-term evidence also contributed, and the Irish papers added explanatory information on how VWs function.

#### Implications for delivering patient care

Patient selection processes should be coherent with the aims of the VW and its common standards agreements. Professionals’ perceptions that the VW is prioritising the ‘right’ patients—taking an acceptable stance on risk of harm and likelihood of benefit—may be important for their trust and motivation. Conversely, the benefits of working in the VW may become less clear if patient selection is ineffective [16, 17, 32, 33, 45, 47].

The most appropriate member of the VW ensures that the patient and/or caregiver are involved in the development of the management plan through a shared decision-making process. The VW coordinator ensures that the wider team is involved in refining and delivering it [16, 28, 33, 47]. The VW can be a supportive learning environment that facilitates this way of working—however, this could be threatened if key team members cannot maintain regular communication necessary for integrated case management [17, 47].

VWs aim to stabilise people living with frailty and mitigate future risk by facilitating timely, proactive interventions [6, 15–17, 28, 32, 33, 36, 39, 41, 44, 46, 47, 49], and to provide as-needed acute care for crises [6, 16, 44, 49]. Depending on the VW model, there is greater emphasis on one or other type of care. Some VWs may have insufficient capacity or time to stabilise patients and rely on GPs to continue the treatment plan, which requires mechanisms ensuring good continuity of care at discharge from the VW [6, 30, 49](Midlothian).

### CMOC Theme 3: patient and caregiver experience (Table 4)

Theme 3 is concerned with patient and caregiver experience, and CMOCs comprise improved communication, at home instead of hospital and caregiver experience. Evidence relating to patient and caregiver experience was limited overall. These CMOCs are derived from evidence from both short-term and longer-term VWs. CMOCs could be directly (*) or indirectly (#) applicable to multidisciplinary non-frailty VWs.

**Table 4:** Summary of CMOCs for Theme 3: patient and caregiver experience

CMOC and sources	CMOC descriptions for Theme 3
CMOC10: improved communication* Sources: 10 documents [15, 16, 28, 34, 44, 47, 48, 50–52], one clinician, three PPI contributors (3/10 short-term, 5/10 non-frailty, 1/10 Ireland)	VW processes enable effective communication and information sharing with the patient/caregiver and provide a route to make contact out of usual working hours. Most VWs do not provide 24-hour cover, but alert systems notify the VW if patients attend emergency care/out-of-hours services [15, 47] [Context]. Time to seek and receive assistance is reduced through enhanced contact mechanisms. Consistent access to a known staff member (the VW co-ordinator) reassures the patient/caregiver [15, 28, 48, 52]. The VW team is well informed, which facilitates responsive treatment [Mechanism]. Improved communication could boost patient and caregiver satisfaction and confidence in managing at home. Anxiety may be reduced through increased awareness of the support in place [Outcome].
**CMOC11: At home instead of hospital** ^**#**^ Sources: 10 documents [16, 28, 29, 32, 33, 35, 44, 46–48], two PPI contributors (4/10 short-term, 4/10 non-frailty, 2/10 Ireland)	Medical crises in people with frailty may be alleviated by intervention, but extended or repeated stays in hospital can have negative health and wellbeing consequences for patients, their family or caregiver(s) [44–46] [PPI]. Usually, patients and caregivers who feel comfortable and secure at home prefer to avoid being in hospital [Context]. The VW facilitates integrated case management for people with frailty, so that appropriate and timely interventions can be delivered at home. Patients and caregivers feel supported and safe [Mechanism]. Remaining in a familiar environment can enable patients and caregivers to maintain existing routines, e.g. physical activity and social support. The disruption of hospitalisation is avoided which may contribute positively to health/wellbeing [Outcome].
CMOC12: caregiver experience* Sources: 5 documents [16, 32, 40, 44, 48], one PPI contributor (2/5 short-term, 3/5 non-frailty, 1/5 Ireland)	Patients and caregivers have practical, informational, and emotional support needs and may find it challenging to navigate complicated healthcare systems [Context]. When caregivers are included in VW communication and shared decision-making, the VW gains insight on the patient’s situation and on patient and caregiver needs. The caregiver feels supported by the VW and valued and listened to [Mechanism]. Involvement in proactive care planning increases caregiver confidence in continuing to manage after patient discharge from the VW, and caregiver burden and stress is reduced [Outcome].

#### Implications for patient and caregivers’ experience

VWs should aim to include the patient and/or their caregiver in decision-making without over-burdening them. Improved communication between the patient/caregiver and the VW, via a known point of contact (e.g. a well-informed, reliable co-ordinator), is expected to be reassuring [15, 28, 48, 52]. It is important to have clear communication on discharge and its timing [16, 44].

Caregivers and patients ideally feel more confident because of VW intervention [16, 48]. However, revoking VW support at discharge may result in increased anxiety, especially if the patient or caregiver does not feel well equipped by the VW to continue at home, or proactive care is not established [44]. Ideally, patients feel empowered to manage [33, 38, 46], but conversely, if VW input means patients feel less enabled, they could lose confidence, potentially increasing stress for both patients and caregivers [33].

Effective continuity of care with primary care is important at discharge for the patient/caregivers to regain confidence living outside the VW [15, 16, 47]. Communication with the GP should support continuation of the management plan, otherwise, patients and caregivers may be left with uncertainty and heightened anxiety [44].

In some cases, the home environment may not be safe, and hospital may be more suitable [44]. It may be that caregivers are unable to take on additional responsibilities, for example, for patients experiencing delirium or other frailty crises, or the home setting is unsafe for delivering acute interventions [44].

## Discussion

This rapid realist review drew from 28 documents and the experiences of clinicians and PPI contributors to identify different VW models operating in the UK. Evidence from all models, including some from Ireland, were used to explain how multidisciplinary VWs can be effective.

In a field where what constitutes a VW is uncertain, we refined, with stakeholders, a definition of VWs as a service delivery model in which an MDT meets and plans patient care remote from the patient. The VW co-ordinates multidimensional interventions at home for people with frailty who are acutely unwell (at a high risk of crisis or in-crisis).

### Summary of findings

We identified two main VW models, which differ in their aims, duration and patients admitted, but in practice, show overlap. Longer-term VWs provide proactive treatment to stabilise the medical and functional status of people living with frailty and reduce the risk of a crisis in people at high risk of deterioration. Short-term VWs (1–21 days) provide acute reactive care to people with frailty already in-crisis, and, if time, start proactive care before discharge to GP care. However, although service models may be different, their populations are closely related: frailty is a continuum, and a minor event can ‘tip’ somebody at high risk of a crisis into crisis. Both models treat patients who would otherwise be in hospital, but at different stages: one seeks to prevent the crisis, and the other provides acute reactive care outside of hospital.

Both models treat people with frailty who are acutely unwell, and each requires a remote MDT to plan patient care. MDT composition will be similar, and appropriate for the VW population, and VW co-ordinators are needed in each. The two models have contributed complementary information to the findings of this review. There are differences in the selection of patients and in post-discharge care: longer-term VWs can discharge to standard primary care as their patient would be stable. For short-term VWs, more responsibility would be passed to primary care because of the need to start/continue proactive care.

Fundamental to VW functioning are key building blocks with their underlying mechanisms. These comprise robust information sharing and common standards agreements that the teams can understand and work within; multidisciplinary teamwork, featuring remote MDT decision-making meetings alongside in-person care; and effective co-ordination, with links to external services (such as out-of-hours). Also important are good relationships within the VW, in-person contact between staff and patients, and involvement and inclusion of patients and caregivers.

Pertinent mechanisms relate to the motivation of professionals to work together and their ability to do so. Ideally, the VW operates as a ‘team-of-teams’ providing mutual support, trust in shared goals and benefit from reciprocal learning. Perceptions of patient safety and benefit, starting small and taking time to introduce formal agreements and learn new ways of working may be necessary for professionals to ‘buy in’ to the VW model. Also essential is good communication between patients, caregivers and staff, and enabling them to feel safe at home and empowered to manage their own care.

Ideally, the VW components combine to ensure the VW can deliver timely interventions to people with frailty who are acutely unwell. However, VWs do not usually provide 24-hour cover. For some people with frailty, crises have an impact on the caregivers who must take on extra responsibility, particularly outside of VW operating hours. Caregivers may feel unable to cope at home with frailty crises (especially incidents of delirium), leading to stress and risk of burnout or patient hospitalisation. VWs may not necessarily be the best arrangement for every situation—acute care in hospital may be required.

### Whole system context

Delivery of VWs for people with frailty should be considered in a whole system context, including transfers of care into and out of the VW.

In longer-term VWs, patients are mainly referred from primary care, following set criteria. In short-term VWs, referrals are likely urgent, and may be from primary care, emergency services or early discharge from hospital. Before reaching a crisis, patients with frailty may have been treated in the community to prevent deterioration, possibly under GP-managed schemes.

Timings and arrangements for discharge to GP care differ: in longer-term VWs, discharge is when the MDT determines patients are stable following proactive care; the co-ordinator arranges good continuity of care. In short-term VWs, discharge may occur when acute events have been resolved; CGA may have been initiated in the VW, but there is insufficient time to establish proactive care. Effective continuity of care on discharge to primary care therefore becomes essential.

Increasing prevalence of frailty would confer greater demand for VW admissions and, potentially, re-admissions if people with frailty are not stabilised. This means that short-term VWs alone may not be sustainable, but they can form part of frailty management in the whole system. There is urgent need for evaluations of short-term VWs. If proactive VWs can prevent crises in people with frailty, they could improve patient outcomes [4], but their cost-effectiveness has not yet been demonstrated.

Rather than an either-or approach to the VW models, it may be that a combination is optimal, particularly in view of the closeness of the two states—high risk of crisis and in-crisis. One study reported such a combined model, comprising a longer-term VW, urgent care, and a care home [6]. Future work could explore a combined approach to acute reactive care and proactive care; for example, with red/amber/green wards within one VW, sharing the same staff and MDT.

### Applicability to non-frailty multidisciplinary VWs

About 60% of the documents describe VWs for people with frailty, but many of the findings (CMOCs) can be applied directly to multidisciplinary VWs for other complex conditions. The specific disciplines and interventions involved would vary, but the underlying mechanisms may be transferable.

### Cost implications

All VWs require investment of resources, which could be offset if VWs are effective in improving decision-making, reducing unplanned or prolonged hospital admission, and minimising duplication of effort between care providers. Cost implications of different VWs models would vary, particularly for staffing and length of stay in the VW, possibly balanced by treatment continuity after discharge. The effectiveness of VWs to mitigate hospitalisation may be highly contingent on resources being available elsewhere (e.g. domiciliary care workers). Cost-effectiveness research should take a broad perspective, including quality of life and costs for caregivers.

Improved use of limited capacity in both hospital and community care is a driver for VW implementation in the NHS [42], recognising that current reactive and hospital-centric care pathways are unsustainable. A more proactive system of care is required [46]. Improvements in frailty management could be cost saving at the system level if people can be reached before a crisis and are better supported to manage at home.

### Comparison with other work

Existing systematic reviews of effectiveness of VWs are limited [24] are restricted to RCTs (of which there are few for frailty VWs), and do not answer questions about how and why VWs are effective. In contrast, this review draws on a range of document types, including grey literature, to answer these questions. Our work may complement systematic reviews of RCTs of community-based complex interventions, which use techniques such as component network meta-analysis to determine components of importance [56].

In December 2021, NHSE produced guidance to introduce ‘virtual wards’ for patients with ‘acute exacerbations of conditions related to frailty’ [19]. Recent work has also focused on short-term VWs for acute care: a rapid evidence synthesis of systematic reviews of acute VWs, hospital-at-home and remote monitoring, across all countries [57], and the British Geriatrics Society’s position paper on VWs for older people with frailty [58]. Our review included a broader range of VW models and was not limited to the more topical short-term VWs, which allowed us to draw on evidence that transcends the type of model.

This rapid realist review is the first to explore how, why, and for whom VWs may deliver effective frailty interventions. The findings show similarities with that of a larger realist synthesis on inter-organisational healthcare, which reports that collaborative leadership ‘works’ when there is trust between the parties involved, faith in the proposed model of care, and confidence in its ways of working [59].

### Strength and limitations of our work

The review explores underlying mechanisms for VWs. We followed RAMESES standards and involved clinicians and PPI stakeholders. However, we were unable to recruit patients with lived experience of a frailty VW, and perspectives on the caregiver experience were limited. Our original intention to hold a large stakeholder consultation exercise was impeded by the COVID-19 pandemic and scheduling limitations.

This rapid realist review focused primarily on the UK, so is directly relevant to current NHS practice, but does draw on evidence from Ireland that has a similar, but slightly different healthcare system to the UK, alongside a similar demographic. This Irish evidence usefully clarified some operational details of frailty VWs, but no aspects of the CMOCs were solely reliant on evidence from Ireland.

Most evidence came from before the COVID-19 pandemic and periods when the UK health system was different in structures and pressures. The role and expectation of technology has changed rapidly but this was not captured in most included documents.

We did not formally appraise the rigour of included documents. We consider its impact on our findings minimal as the synthesis generates hypotheses rather than evaluating effectiveness or testing theories.

## Conclusions

This rapid realist review outlines different VW models for people with frailty. Some findings can be applied to multidisciplinary VWs for other complex conditions. Our work could inform future decisions regarding service planning, evaluation and implementation of multidisciplinary VWs. There is currently insufficient evidence on the sustainability of VW models, experiences of caregivers, or the impact of social inequalities, all of which should be examined further.

Establishing a VW should involve formal collaboration agreements and starting small when adopting new ways of working. Perceptions of patient safety and benefit are important to maintain professionals’ ‘buy-in’ to the VW model. Time and resource should be planned into professionals’ work schedules.

The risk of caregiver stress, anxiety or burnout in some situations should be considered, especially after hours when VWs may not provide support. For some patients, hospital with 24-hour care could remain the most appropriate setting.

Sustainable frailty management requires that people with frailty are identified before reaching a crisis, and receive proactive care, monitoring, and support to self-manage, thereby preventing crisis situations and associated negative outcomes for patient, caregiver and the healthcare system. Reactive short-term VWs may be useful as a safety net for people who do fall into crisis. A whole system approach to effective frailty management is necessary, with attention to continuity of care including VW referral and discharge experiences. Our findings indicate a possible role for a combination of VW models.

Optimal implementation and delivery of multidisciplinary VWs could potentially improve quality of life for patients and caregivers, whilst alleviating resource demands of frailty management for the healthcare system.

## Supplementary Material

aa-23-0659-File002_afae039
